# Comparison of the accuracy of axial length measurement by different imaging methods in Sprague Dawley rats

**DOI:** 10.3389/fnins.2022.1106904

**Published:** 2023-01-06

**Authors:** Yajun Wu, Xiangdong Luo, Yuliang Feng, Jiasong Yang, Hua Fan, Xiaobo Cen, Wensheng Li

**Affiliations:** ^1^Aier School of Ophthalmology, Central South University, Changsha, Hunan, China; ^2^Department of Ophthalmology, Shanghai Aier Eye Ophthalmology Hospital, Shanghai, China; ^3^Shanghai Aier Eye Institute, Shanghai, China; ^4^Department of Ophthalmology, Xiamen Eye Center of Xiamen University, Xiamen, Fujian, China; ^5^WestChina-Frontier PharmaTech Co., Ltd., Chengdu, Sichuan, China

**Keywords:** Quantel A-B scan, OD-1 A scan, vernier caliper, axial length, measuring method

## Abstract

**Background:**

Obtaining accurate axial length (AL) is very important for the establishment of animal models of myopia. The purpose of this study is to compare the accuracy of Quantel A-B scan, OD-1 A scan, and vernier caliper in measuring AL in Sprague Dawley (SD) rats.

**Methods:**

In total, 60 5-week-old SD rats were divided into female rat group (*n* = 30) and male rat group (*n* = 30). Quantel A-B scan and OD-1 A scan were, respectively, used to measure the AL of both eyes of each living rat, and vernier caliper was used to measure the anterior-posterior diameter of each rat’s eyeball. Besides, the correlation between refractive error (RE) and AL measured by different instruments was evaluated, and the accuracy of the three measurement methods was compared according to gender and left/right eyes.

**Results:**

There were significant differences in AL and diopter of SD rats at the same age (*p* < 0.05). the AL of male rats was greater than that of female rats, while diopter (D) was the opposite; There was no significant difference in AL and D between left and right eyes in the same SD rats (*p* > 0.05); There were statistical differences among the three measurement methods (*p* < 0.05), AL measured by vernier caliper was the largest, followed by Quantel A-B scan, OD-1 A scan; Difference in AL between male and female was not statistically significant between the results obtained by Quantel A-B scan and vernier caliper (*p* > 0.05), but there were statistically significant differences between the other two measurement methods (*p* < 0.05).

**Conclusion:**

Sex is the influencing factor of AL and RE. Imaging measurement can accurately measure the AL in living small rodents. Compared with OD-1 A scan, Quantel A-B scan may be more accurate.

## Introduction

Myopia is now a puzzle for public health worldwide (Ramamurthy et al., 2015) with a prevalence that continues to rise. It is predicted that, by 2050, the number of people with myopia will reach as high as 4.95 billion, nearly half of the global population (Holden et al., 2016). At present, the characteristics of myopia include low age and advanced development, with more and more young people suffering from myopia. In East Asia in particular (He et al., 2015), myopia is more common, and, indeed, 80–90% of young people aged 14–35 in China suffer from myopia (Ma et al., 2021). According to statistics, the myopia rate of 12-year-old children in Hong Kong is as high as 61%, and that of adults is as high as 41.1% (Mak et al., 2018). The myopia rate in Taiwan’s 18–24-year-old youth conscription group is as high as 86% (Lee et al., 2013). Moreover, high myopia (HM) often causes myopic choroidal neovascularization (mCNV), complicated glaucoma, cataract, macular hole (MH), and other blinding complications (Wu et al., 2016), which result in a heavy economic burden to families and society. Statistically, the annual productivity loss as a result of myopia in China is as high as US $26.3 billion (Ma et al., 2022), whereas the global productivity loss caused by myopia alone was as high as US $244 billion in 2015 (Naidoo et al., 2019). These values will increase as the prevalence of myopia increases in the population. Therefore, myopia prevention and control is urgent.

Obviously, understanding the mechanisms of myopia occurrence and development is the key to slowing down the rapid rise of its global prevalence effectively (Baird et al., 2020), and the emergence of animal models of myopia is very important to the research of myopia-related mechanisms. At present, the animals used widely in myopia research include monkeys (Zhu et al., 2013), chickens (Liu et al., 2020), guinea pigs (Zhou et al., 2020), rats (Chen et al., 2021), and mice (Lin et al., 2021). The two primary animal models are form-deprivation myopia (FDM) and lens-induced myopia (LIM), respectively. The refractive status of the eye is determined mainly by the axial length (AL) or refractive error (RE) and the matching relationship between them. In fact, the AL is related closely to the RE (Chamberlain et al., 2021). Myopia is not a simple RE, but is often accompanied by axial growth, retinal and scleral thinning, and other pathological changes (Baird et al., 2020), and the animal models of myopia confirmed these above pathological changes clearly (Troilo et al., 2019). Therefore, AL is also one of the important parameters to evaluate myopia, which is why animal models usually judge whether myopia induction is successful according to the results of diopter (D) and AL at the same time.

Although there are many kinds of animals that can be selected for myopia models, there are differences in the physiological structure of the eyes of different animals, which leads to different choices of different animals in establishing myopia models (Schaeffel and Feldkaemper, 2015). The structure of the chicken eyeball is quite different from that of human eye (Glasser and Howland, 1995), and the accuracy of the conclusion of myopia research in chicken models is worthy of further confirmation. The eyes of monkeys have macular fovea, the physiological structure of which is similar to that of humans, and the instruments available for human eyes can be applied directly to monkeys, which therefore makes the monkey eye an ideal model for myopia. However, monkeys are expensive, have a long experimental period, and are difficult to domesticate, therefore, few research teams can use monkeys to conduct large-sample myopia studies. Small rodents, rats, mice, and guinea pigs, are cheap and reproduce quickly and in large numbers. Their eyeball development process is also similar to that of humans. They all have hyperopia reserves in rodents, and gradually face up with age, which can be better used in myopia research.

However, although small rodents have been used widely in myopia research, their eyeballs are small and it is difficult to measure ophthalmic parameters. Although, at present, optical coherence tomography (OCT) can be used successfully to measure the living eyeball parameters of small animals, such as mice (Zhou et al., 2008), the cost of measuring AL is high and it OCT is inconvenient to use. For animals with AL > 15 mm that can be measured by conventional ophthalmology A-scan, it is difficult to measure small animals, such as rats, and guinea pigs, with small eyeballs accurately. Even if measurements can be made, manual mode should be used, which has certain measurement errors. A-B scan (Quantel, Les Ulis, France) has been used to measure the AL of guinea pigs (Dong et al., 2019). Compared with OCT or B scan, it is simple to operate and is a portable instrument, but it cannot be measured automatically on small animals directly. The manual measurement mode is required, and there is error. OD-1 A scan (Kaixin, China) developed an A-scan measurement mode independently, which can manually measure the AL of small animals such as, rats, and guinea pigs, manually.

So far, there has been no comparative study on the AL measurement methods of the AL of small animals such as rats. Therefore, the purpose of this study is to compare the AL of Sprague Dawley (SD) rats measured by Quantel A-B scan and Kaixin OD-1 A scan with the AL measured by vernier caliper after anatomy, and also to explore if there are differences in the AL of SD rats with different of sexes and eyes, so as to provide a reliable basis for the study of myopia in small rodents.

## Materials and methods

### Animals

A total of 60 SD rats [Purchased from Beijing Weitong Lihua Laboratory Animal Technology Co., Ltd., China. Production License No: SCXK (Beijing) 2021-0011] were included in this experiment, male/female.

Inclusion criteria: (1) 5-week old SD rats, with age difference ≤ 1 week; (2) The body weight of females was 180–220 g and males 300–400 g, and the individual weight was within the mean ± 20%; (3) Eye condition is good, cornea, lens, vitreous eye refractive system is normal, no turbidity, inflammation. Exclusion criteria: (1) SD rats with poor ocular and systemic conditions, such as eye trauma, corneal opacity, and poor mental state; (2) SD rats with irritable temperament and difficult to accept A-scan and retinoscopy under non-general anesthesia; (3) Rats whose weight and age exceed the inclusion criteria.

All SD rats were adapted to the environment for 3–5 days before the experiment, and healthy rats were selected as the test animals. According to gender, All SD rats were divided into female group (*n* = 30) and male group (*n* = 30). All animals were housed in a PP-4 mouse cage (L × W × H: 400 mm × 250 mm × 200 mm) in WestChina-Frontier PharmaTech Co., Ltd., Chengdu, Sichuan, China, with five animals per cage. If the animals were abnormal, they were housed in a single cage. The feeding environment was in accordance with the national standard of the People’s Republic of China GB14925-2010, with room temperature of 20–26°C (daily temperature difference ≤ 4°C), relative humidity of 40–70%, artificial lighting, alternating light and dark day and night for 12/12 h, and all animals were free to eat and drink.

This study has passed the ethical approval of the Committee of WestChina-Frontier PharmaTech Co., Ltd. with the ethical approval number of IACUC- SW-S2022007-P001-01.

### Retinoscopy

Fix the rats, completely expose the cornea, and measure the refractive state with the red strip Optometry (Suzhou 66 Vision Technology Co., Ltd., China). Under dark room conditions, SD rats were dripped with 5 g/l compound tropicamide eye drops to dilate their pupils and paralyze the ciliary muscles, once every 5 min, at least three consecutive times. After 30 min, the experimenter grabbed the rats to expose the examined eyes. An experienced optometrist performed retinoscopy and optometry on all rats at a working distance of 50 cm, and performed retinoscopy on the horizontal and vertical meridians at an interval of 0.5 D, respectively. The astigmatism will be calculated by half equivalent spherical lens (Zhou et al., 2007).

### Quantel A-B scan (Quantel, Les Ulis, France) measures AL

All SD rats were subjected to superficial anesthesia by dropping 4 g/l obucaine hydrochloride eye drops on the eye surface. The operation was repeated 2–3 times with an interval of 5 min each time, and the corneal reflex disappeared as the standard. Open the instrument, input the rat number, select the eye type (right then left), select the A-scan manual measurement mode, anterior chamber and vitreous set to 1,557.5 ms^–1^, lens set to 17,233.3 ms^–1^ (Jiang et al., 2014). The probe was pointed at the pupil area of the rat, and the cornea was vertically touched (without compression). The clear and stable waveform was taken as the determined image. The value of the waveform of the anterior interface of the retina is read as AL.

### OD-1 A scan (Kaixin, China) measures AL

All SD rats were surface anesthetized with 4 g/l obucaine hydrochloride eye drops. The operation was repeated 2–3 times with an interval of 5 min. The disappearance of corneal reflex was taken as the standard. Turn on the instrument, enter the rat number, select the eye type (right first, then left), select the mode of manual measurement of AL in small animals. aim the probe at the center of the rat pupil, touch the cornea vertically (without pressing the cornea), take a clear and stable waveform as the determined image, read and record the scale value of the anterior interface of the retina wave peak, measure three times for each eye, and take the average value, accurate to 0.01 mm.

### The vernier caliper (Shanghai Measuring Tools Co., Ltd., China) measures the AL

Sprague Dawley (SD) rats were anesthetized with 3% sodium pentobarbital sodium (50 mg/kg, intraperitoneal injection) and euthanized by bleeding of abdominal aorta. quickly remove the complete eyeballs in the super clean workbench, measure the anterior and posterior diameters of the binocular axes with a vernier caliper (Distance from corneal apex to optic disc of eyeball with clean connective tissue and optic nerve removed), accurate to 0.02 mm. The axial values were read and recorded, and the average values were measured three times for each eye.

### Statistical analysis

Statistical software SPSS25.0 (IBM Corp., Armonk, NY, USA) was used for analysis. Measurement data in this study were described as mean ± SD. Levene test was used to test the homogeneity of variance for axial index. When the variance was homogeneity (*P* > 0.05), one-way analysis of variance (ANOVA) was used for statistical test. When the variance was uneven (*P* ≤ 0.05), Kruskal-Wallis H rank sum test was used for statistical analysis. Paired-*t*-test was used for comparison of AL between left and right eyes, independent sample-*t* test was used for pairwise comparison between different groups, and ANOVA was used for comparison among three groups. *P* < 0.05 was considered statistically significant. Pearson correlation analysis was used to analyze the correlation between AL and D.

## Results

### Comparison of AL results from three different measurement methods

**FIGURE 1:**
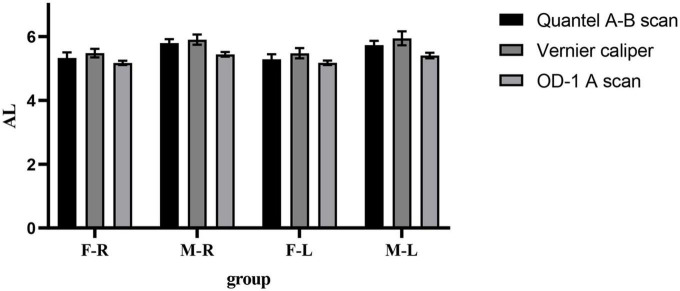
Comparison results of the mean values of three AL measurement methods. F-R, female group right eye; M-R, male group right eye; F-L, female group left eye; M-L, male group left eye; AL, axial length.

**FIGURE 2:**
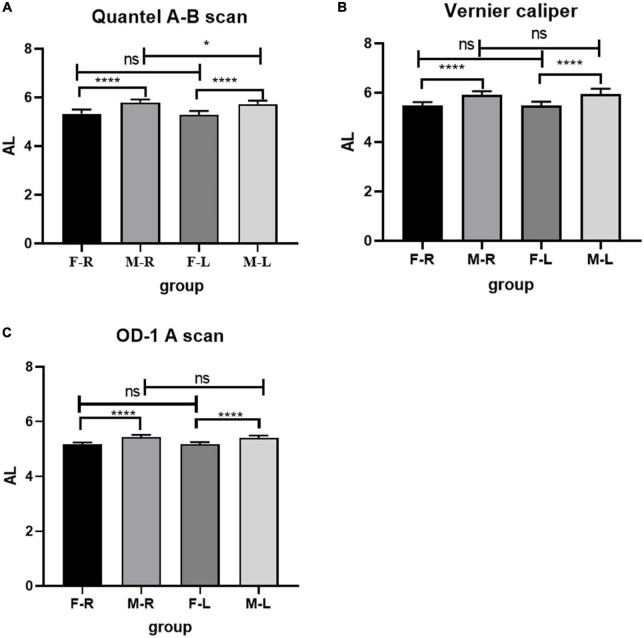
Comparison of AL in Sprague Dawley (SD) rats of different sex and eye types. **(A)** AL results from Quantel A-B Scan. **(B)** AL results from Vernier caliper. **(C)** AL results from OD-1 A scan. F-R, female group right eye; M-R, male group right eye; F-L, female group left eye; M-L, male group left eye; AL, axial length; ns indicates no statistical significance; *p < 0.05; ****p < 0.01.

In the male and female rat groups, the AL measured by vernier caliper of the left and right eyes was greater than that measured by Quantel A-B scan, and the result value of OD-1 A scan was the smallest (as shown in Figure 1). The AL measured by the three methods was statistically significant (*p* < 0.05). Taking the eye type as the variable, the Quantel A-B scan measurement results in the female rat group showed that there was a statistical difference in the AL of the left and right eyes (*t* = 2.050, *p* = 0.045; as shown in Figure 2A), and the other measurement results showed that there was no statistical difference in the AL of the left and right eyes of SD rats of the same sex (*p* < 0.05; as shown in Figures 2B, C). In the same eye, the AL of male rats was significantly higher than that of female rats. See Table 1 for the details.

**TABLE 1:** Axial length (AL) of left and right eyes in each group.

Group	Male (*n* = 30)		T	P	Female (*n* = 30)		T	P
	R (mm)	L (mm)			R (mm)	L (mm)		
Quantel A-B scan	5.798 ± 0.122	5.729 ± 0.140	0.879	0.383	5.324 ± 0.176	5.286 ± 0.158	2.050	0.045
Vernier caliper	5.902 ± 0.160	5.942 ± 0.218	0.123	0.903	5.481 ± 0.135	5.476 ± 0.159	−0.816	0.418
OD-1 A scan	5.441 ± 0.073	5.403 ± 0.088	−0.257	0.798	5.173 ± 0.068	5.178 ± 0.072	1.831	0.072
F	114.629	88.522			39.555	37.116		
P	0.000	0.000			0.000	0.000		

R, right eye; L, left eye; T, *t*-value; P, *p*-value; F, *f*-value (*p* < 0.05) difference was statistically significant.

### Results of RE

**FIGURE 3:**
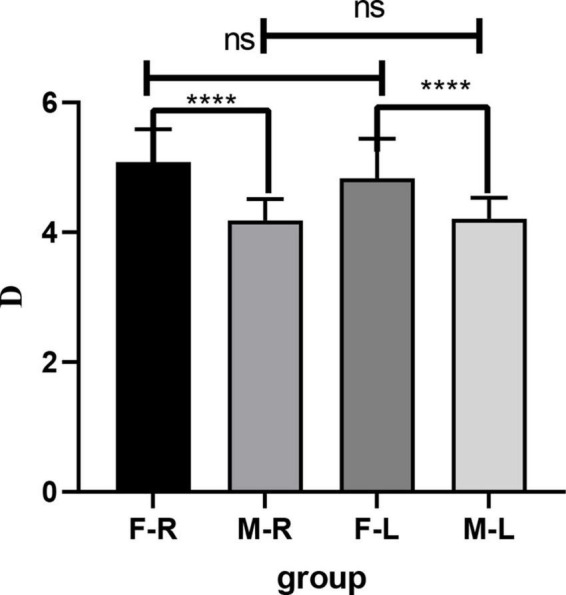
Comparison of RE in Sprague Dawley (SD) rats of different sexes and eye types. F-R, female group right eye; M-R, male group right eye; F-L, female group left eye; M-L, male group left eye; RE, refractive errors; D, diopter; ns indicates no statistical significance; ****p < 0.01.

The RE of the female group was higher than that of the male group (F-R: 5.083 ± 0.506D; M-R: 4.183 ± 0.328D; F-L: 4.833 ± 0.610D; M-L: 4.208 ± 0.322D), the difference was statistically significant (*p* < 0.05), and there was no significant difference in RE between the left and right eyes (*p* > 0.05). See Figure 3 for details.

### Comparison of AL difference between male and female rats by three different measurement methods

**FIGURE 4:**
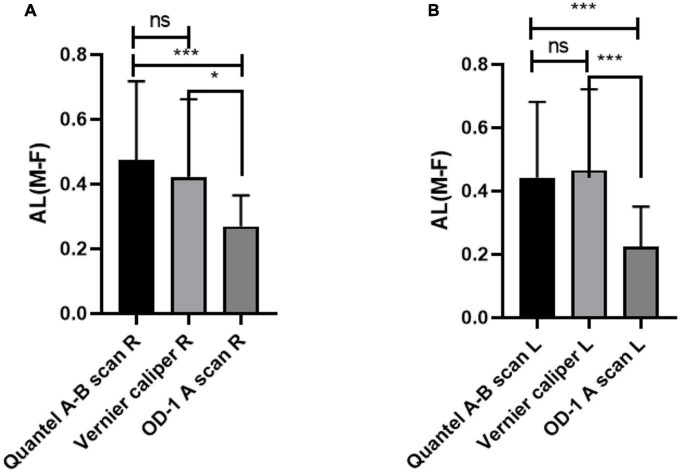
Comparison of AL difference between male and female rats by three different measurement methods. **(A)** AL difference in right eye between male and female rats. **(B)** AL difference in left eye between male and female rats. AL, axial length; R, right eye; L, left eye; M-F, AL difference between male and female rats; ns indicates no statistical significance; *p < 0.05; ***p < 0.01.

The comparison of AL difference between male and female rats shows that there is no significant difference in the measurement results between Quantel A-B scan and Vernier caliper, while there are statistical differences between Quantel A-B scan and OD-1 A scan, Vernier caliper and OD-1 A scan. The details are shown in Figure 4.

### Correlation analysis between AL and D

**FIGURE 5:**
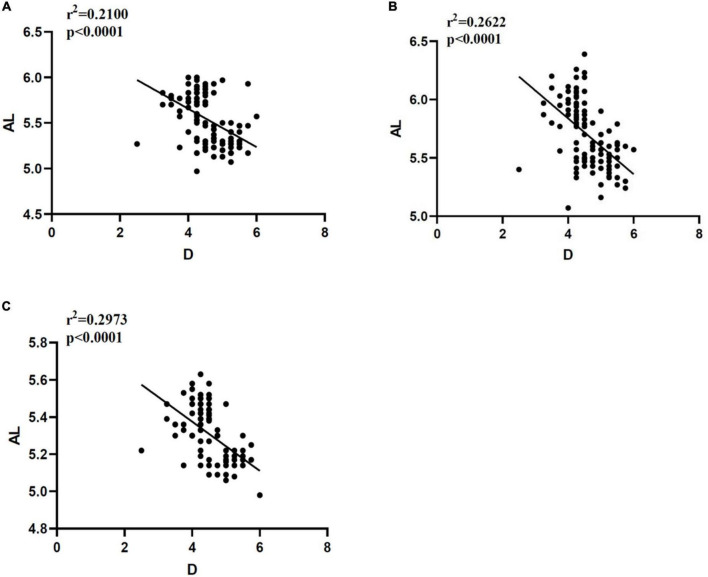
Correlation analysis between AL and D measured in different ways. **(A)** Correlation between AL and D measured by Quantel A-B scan. **(B)** Correlation between AL and D measured by Vernier caliper. **(C)** Correlation between AL and D measured by OD-1 A scan. AL, axial length; D, diopter; r2, Pearson correlation coefficient; *p* < 0.0001 indicates that there is great statistical significance.

The AL results measured in three different ways have negative correlation with D (*p* < 0.0001). The Pearson correlation coefficient *r*^2^ shows that Quantel A-B scan is 0.2100, Vernier caliper is 0.2622, and OD-1 A scan is 0.2973. The details are shown in Figure 5.

## Discussion

The commonly used AL measurement methods in clinical practice mainly include mainly the optical method and ultrasonic bioassay, the former is mainly intraocular lens-master (IOL-master) (Roy et al., 2012) and OCT, whereas the latter is mainly A-scan. However, the optical method is used more widely because of its higher measurement accuracy than the ultrasonic method, and is considered as the gold standard of AL measurement (Goto et al., 2020a). However, the optical method has high requirements on the refractive stroma. Once the lens, cornea, and other refractive stroma, are seriously cloudy, AL cannot be obtained accurately, whereas the ultrasound method is not subject to this limitation. Thus, at present, both methods are often used for AL measurement. In addition, AL measured by the optical method refers to the distance from corneal apex to retinal pigment epithelium (RPE) cells, whereas AL measured by the ultrasonic method refers to the distance from corneal apex to inner limiting membrane (ILM), so the result of the latter is slightly smaller than that of the former (Goto et al., 2020b).

However, although the accuracy of AL measurement by A-scan is not as high as that by the optical method, it is more convenient to apply to small animals. Both OCT and IOL Master are difficult to coordinate with the measurement of animals, which not only needs to be performed under general anesthesia, but also tends to increase the mortality of experimental animals, and it is difficult to support microaxial detection using this instrument. Therefore, A-scan is more suitable for the measurement of AL in small animals. In fact, in most studies of small rodent myopia models, AL was measured by A-scan (Tian L. et al., 2021; Yang et al., 2021; Yu et al., 2021). This is the reason why two kinds of A-scan were selected to measure the AL of rats in this study.

Axial length (AL) is defined as the length of the anterior and posterior diameters of the eyeball. For this reason, we chose vernier caliper to measure the anterior posterior diameter of the eyeball of the intact rat after removing the conjunctival tissue as the gold standard of AL. Because the thickness of all tissues (choroid and sclera) after retinal ILM is included, the measurement result will be greater than that of A-scan. Consistent with our results, the AL results measured by the vernier calipers of all rats were greater than those of the other two groups of A-scan.

Moreover, because the results of RE tend to be synchronized with the increase of AL (Tian T. et al., 2021), there is a negative correlation between them (Jiang et al., 2009), and the AL of infant female rats is smaller than that of male rats of the same age, therefore we examined the AL and RE results of male and female rats at 5 weeks of age. Finally, we found that the AL of male rats was greater than that of female rats, and the difference was significant.

Furthermore, the correlation between AL and D in each group was analyzed and all three AL values had significant negative correlation with D. In addition, we compared the AL difference between male and female rats measured by three different ocular axis measurement methods, and found that there was no significant difference between Quantel A-B scan and vernier caliper, although there was a statistical difference between OD-1 A scan and the other two methods. These indicate that Quantel A-B scan might be more accurate for AL measurement of living small rodents, although the accuracy of this instrument is only 0.1 mm, whereas OD-1 A scan can be accurate to 0.01 mm. In addition, the price of the latter is only 1/10 of the former, which might therefore be a better choice for laboratories under poor economic conditions. Although the image measurement results are not as accurate as the anatomical results, they are sufficient for measuring the AL of small experimental animals.

As far as we know, this is the first study comparing the AL values of SD rats measured by an anatomical method and an imaging method, and the results have certain significance for the study of myopia in small animals. Unlike human eye measurements, the AL of small animals is too small to be measured accurately, although, admittedly, the AL measurement methods for human eyes are improving constantly. Among them, swept source optical coherence tomography (SS-OCT) is considered to be the most accurate AL measurement method at present (Vounotrypidis et al., 2019). Huang et al. (2019) compared the AL values measured by three kinds of SS-OCT with those measured by partial coherence interferometry (PCI), and found that the success rate of AL measurement of SS-OCT biometrics was significantly higher than that of PCI, and so they believed that SS-OCT might become the gold standard for AL measurement. For animal studies, the myopic animal model can meet the acquisition of real AL (anterior posterior diameter of ocular axis), if it is not required to obtain AL data of living animals, we believe that AL measured by vernier caliper can be used as the gold standard of rat AL, which can be accurate to 0.02 mm. However, we recommend A-scan for the acquisition of AL in small living animals.

Our study has the following limitations. First, we did not measure the tissue thickness of the eyeball retina after ILM, so we could not judge accurately the difference between the AL value measured by the two A-scan and the actual eyeball anteroposterior diameter, which requires the assistance of SS-OCT. Unfortunately, we are unable currently to meet this condition; Second, we did not detect the changes of AL and D from young to adult rats to judge the specific impact of changes in the ocular axis on RE, so as to further detect the accuracy of Quantel A-B scan and OD-1 A scan. Third, we chose SD rats as the representative of small animals because they can be used as myopia models. Compared with other common models, such as, mice, chickens, their eyeballs are larger and easier to measure. In fact, we also used these two A-ultrasound to measure AL in guinea pigs, and successfully obtained AL. However, we have not tested mice and chickens, so we cannot determine whether their AL can be obtained by A-scan. We aim to address these limitations in future studies.

## Conclusion

From what we have discussed here, we have confirmed that A-scan can satisfy the acquisition of AL in small animals. Our results of Quantel A-B scan for AL measurement of living small animals might be more reliable, whereas the vernier caliper can obtain the actual anterior posterior diameter of the ocular axis, however, it needs to be obtained after the animals are dissected. With the development of technology, we believe that accurate and economical AL measuring instruments for small animals can be developed in the future, which will provide strong support to relevant myopia research.

## Data availability statement

The original contributions presented in this study are included in the article/supplementary material, further inquiries can be directed to the corresponding authors.

## Ethics statement

The animal study was reviewed and approved by the Committee of WestChina-Frontier PharmaTech Co., Ltd. with the ethical approval number of IACUC- SW-S2022007-p001-01.

## Author contributions

YW and XL were responsible for writing and modifying the manuscripts. YF and JY were responsible for proofreading and revising the language of the manuscript. HF was responsible for data statistics and chart making. WL and XC were responsible for the design of the study and guiding the revision of the manuscript. All authors contributed to the article and approved the submitted version.
